# Estimating the incidence of actionable drug-gene interactions in Japanese patients with major depressive disorder

**DOI:** 10.3389/fpsyt.2025.1542000

**Published:** 2025-03-27

**Authors:** Masakazu Hatano, Masashi Ikeda, Takeo Saito, Masami Miyata, Nakao Iwata, Shigeki Yamada

**Affiliations:** ^1^ Department of Pharmacotherapeutics and Informatics, Fujita Health University School of Medicine, Toyoake, Aichi, Japan; ^2^ Department of Psychiatry, Nagoya University Graduate School of Medicine, Nagoya, Aichi, Japan; ^3^ Department of Psychiatry, Fujita Health University School of Medicine, Toyoake, Aichi, Japan; ^4^ Research Promotion Headquarters Development Office for Genomic Medical Research Center, Fujita Health University School of Medicine, Toyoake, Aichi, Japan

**Keywords:** antidepressive agents, CYP2B6, CYP2C19, CYP2D6, depressive disorder, pharmacogenetics

## Abstract

**Background:**

Although several guidelines provide dosing recommendations for antidepressants based on patients’ genetic information, pharmacogenetic testing for antidepressant use is rarely routinely performed in Japan. To clarify the clinical impact of pharmacogenetic testing, this study estimated the potential drug-gene interactions for first-time antidepressant treatment in Japanese patients with major depressive disorder.

**Methods:**

This study retrospectively included Japanese patients who were registered for depressive episodes (F32, International Classification of Diseases, Tenth Revision) and initiated on antidepressants between July 2022 and March 2023. Antidepressant prescription rates were calculated using a nationwide hospital-based database (Medical Data Vision Co., Ltd). The incidence of actionable drug-gene interactions was estimated by multiplying the first-time prescription rate of each relevant antidepressant by the frequency of its corresponding actionable phenotype.

**Results:**

A total of 3,197 patients were included in the analysis. Escitalopram was the most frequently prescribed antidepressant (18.7%, n = 597), followed by mirtazapine (17.5%, n = 561), and sertraline (15.4%, n = 493). Of the patients receiving their first treatment of major depressive disorder, 56.5% (n = 1,807) were prescribed a drug with actionable pharmacogenetic implications, and 26.4% (n = 844) were estimated to have required actionable therapeutic recommendations. The highest incidence of actionable drug-gene interactions was observed in escitalopram and CYP2C19 pairs (12.4%, n = 398). For sertraline and CYP2C19 or CYP2B6 pairs, the incidence was 11.0% (n = 352). Among all antidepressants, paroxetine had the highest incidence of actionable drug-gene interactions related to CYP2D6 at 1.8% (n = 56); this interaction was rarely observed with other antidepressants (<1%).

**Conclusions:**

We estimated that one in four Japanese patients with major depressive disorder who were prescribed first-time antidepressants had actionable drug-gene interactions. These results suggest that pre-emptive pharmacogenetic testing in the treatment of major depressive disorder could have important clinical implications.

## 1 Introduction

Pharmacogenetics (PGx) aims to identify genetic factors that influence drug efficacy and side effects to optimize treatment for individual patients (1). Pre-emptive PGx testing allows for the prediction of drug responsiveness, enabling dose adjustments or changing to alternative drugs from the beginning of treatment. In Europe, pre-emptive PGx panel testing of 12 genes resulted in a 30% reduction in clinically relevant adverse reactions (2). The Clinical Pharmacogenetics Implementation Consortium (CPIC), an international consortium established to facilitate the clinical implementation of PGx testing, has published guidelines for over 300 drug-gene interactions (DGIs) (3). Furthermore, over the past 20 years, there has been an increase in the inclusion of PGx information in drug labels for US FDA approvals. This trend is particularly noticeable in oncology medications, which comprise roughly half of all new drugs approved with PGx labeling (4). PGx testing has been adopted in clinical practice in Japan. For example, UGT1A1 testing is frequently performed to predict the risk of side effects associated with irinotecan (5, 6). Conversely, PGx-informed prescribing in non-cancer therapeutic areas may involve drugs with lower individual risk but potentially substantial impact at the population level due to high prescription rates. Notably, an Irish study identified antidepressants as the most frequently dispensed drugs among those for which actionable recommendations require direct intervention (7).

Major depressive disorder (MDD) is the most common mental disorder, with a lifetime prevalence of 10.8% (8). Initial treatments for depression include psychotherapy and pharmacotherapy, with antidepressants recommended for moderate to severe cases (9–11). Most antidepressants are metabolized by cytochrome P450 (CYP) enzymes, and CYP gene polymorphisms are known to cause inter-individual differences in pharmacokinetics (12). An example is the drug-gene pair escitalopram and CYP2C19, where poor metabolizers (PMs) were found to exhibit significantly increased escitalopram exposure compared to normal metabolizers (NMs) (13). Similarly, a meta-analysis of genome-wide association studies reported that PMs were associated with an increased risk of side effects, including gastrointestinal, central nervous system, and sexual side effects (14). Based on these findings, CPIC guidelines recommend a 50% reduction in the standard maintenance dose of escitalopram for CYP2C19 PMs (15). Similar actionable recommendations exist for several other antidepressants; nevertheless, routine PGx testing for antidepressants is rarely performed in Japan.

To facilitate pre-emptive PGx testing in MDD pharmacotherapy, it is essential to evaluate its potential clinical impact. It has been estimated that 19.1–23.6% of new prescriptions in primary care involve DGIs, and 5.4–9.1% could necessitate dose adjustments or alternative drug choices (16, 17). However, the impact of PGx testing varies across therapeutic areas, and differences in the frequency of CYP polymorphisms among ethnic groups and drug prescription patterns must also be considered.

In this study, we estimated the actionable DGIs in antidepressant treatment for Japanese patients with MDD. Using a nationwide hospital-based database, we calculated the prescription rates antidepressants associated with actionable PGx for first-time MDD treatment. We then investigated the incidence of drugs that meet the actionable recommendations in the CPIC guidelines based on the phenotype frequency in the Japanese population.

## 2 Materials and methods

### 2.1 Selection of actionable drug-gene interactions

We selected antidepressants and genes for this study based on the CPIC guidelines. Antidepressants were included if they had therapeutic recommendations for at least one phenotype and were approved for use in Japan (15, 18). Actionable DGIs were examined for the following drug-gene pairs: CYP2C19 with escitalopram, sertraline, amitriptyline, imipramine, clomipramine, and trimipramine; CYP2B6 with sertraline; and CYP2D6 with paroxetine, fluvoxamine, venlafaxine, vortioxetine, amitriptyline, imipramine, clomipramine, nortriptyline, and trimipramine.

### 2.2 Nationwide first-time antidepressant prescription rate

Using the nationwide hospital-based database provided by Medical Data Vision Co., Ltd. (MDV) in Japan, we calculated prescription rates for each antidepressant used for first-time treatment in patients with MDD. This database contains anonymized records of approximately 43.4 million patients treated at 477 hospitals which have adopted the Diagnosis Procedure Combination (DPC) system as of April 2023. The DPC is a case-mix patient classification system developed in Japan (19). Diagnoses were coded according to the International Classification of Diseases, Tenth Revision (ICD‐10), and prescribed medications were assigned the Anatomical Therapeutic Chemical (ATC) classification.

This study adopted a new-user design (20). Patients were eligible if they were registered for depressive episodes (F32) according to the ICD-10 and were initiated on antidepressants between July 2022 and March 2023. The date when the antidepressant was first prescribed was set as the index date for this study. Patient inclusion criteria were as follows: an active history of ≥ 180 days prior to the index date in the MDV database; no records of antidepressant use within 180 days prior to the index date; and an age of 18–69 years. The following patients were excluded from this analysis: those prescribed antidepressants for delirium; those prescribed antidepressants by non-psychiatrists; those prescribed antidepressants as needed; and those prescribed multiple antidepressants on the index date.

Antidepressants were defined based on the ATC classification as N06A4 (selective serotonin reuptake inhibitors), N06A5 (serotonin and norepinephrine reuptake inhibitors), and N06A9 (other antidepressants) (**Supplementary Table 1**). We adopted a delirium identification algorithm modified from previous studies, wherein delirium was identified if one of two conditions was met: an ICD-10 code F05 (delirium due to unknown physiological condition) during hospitalization or prescription of at least one antipsychotic (haloperidol, olanzapine, perospirone, quetiapine, or risperidone) between the admission date and the next seven days (21). Patients prescribed antipsychotics during the first two days of hospitalization were considered prevalent users and excluded from the criteria for delirium (washout period). Additionally, patients who were hospitalized for less than three days were also excluded. The study design diagram is illustrated in **Supplementary Figure 1** (22).

### 2.3 Phenotype frequency

The phenotypic frequencies of CYP2C19 and CYP2D6 in the Japanese population were obtained from a meta-analysis of probability estimates by Koopmans et al. (23). Regarding CYP2B6, no study to date has yet to comprehensively evaluate its frequency in the Japanese population; therefore, we calculated the mean phenotype frequency by weighting the sample sizes of three studies (24–26). Ultrarapid and rapid metabolizers were pooled and analyzed collectively as ultrarapid metabolizers (UMs), according to the method described by Koopmans et al. In addition, since sertraline is associated with CYP2C19 and CYP2B6, and amitriptyline with CYP2C19 and CYP2D6, the combined phenotypic frequency considering both phenotypes was estimated by multiplying the individual phenotypic frequencies.

### 2.4 Estimation of potential actionable drug-gene interactions

The incidence of actionable DGIs in first-time pharmacotherapy for MDD in the Japanese population was estimated by multiplying the prescription rate of the relevant antidepressant by the frequency of the corresponding actionable phenotype (7, 17). However, the total number of actionable DGIs could be overestimated if multiple gene interactions were associated with a single antidepressant; therefore, in such cases, the gene with the highest frequency of an actionable phenotype was chosen for the estimation. For sertraline and amitriptyline, clinical recommendations have been provided for combinations of CYP2C19 and CYP2B6 or CYP2D6. Thus, the incidence of actionable DGIs was estimated by multiplying the prescription rate for each drug by the frequency of the corresponding combined actionable phenotype. All data analyses were performed using the R software, version 4.4.1 (The R Foundation for Statistical Computing, https://www.r-project.org/).

## 3 Results

### 3.1 Population characteristics

**Figure 1:**
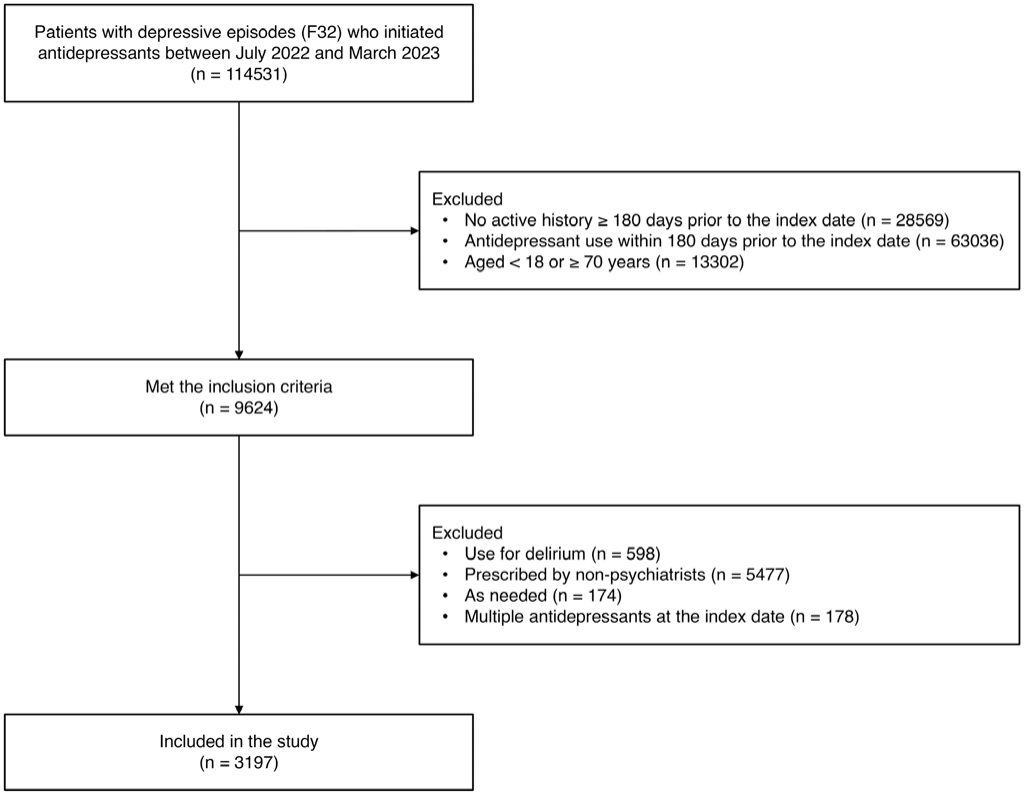
Study flow diagram.

**Figure 2:**
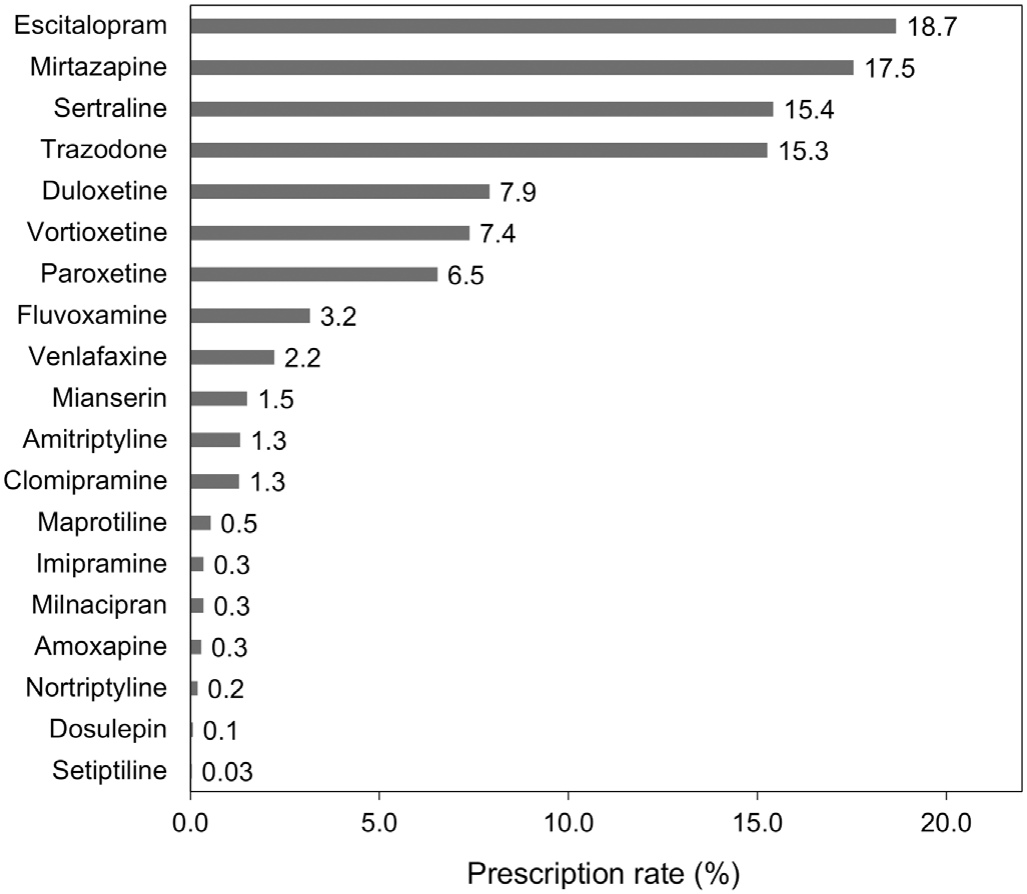
Prescription rates of antidepressants in first-time treatment for major depressive disorder.

The process of patient inclusion and exclusion is outlined in **Figure 1**. We identified 114,531 patients who were diagnosed with a depressive episode and prescribed antidepressants between July 2022 and March 2023 in the MDV database, out of which 9,624 met the inclusion criteria. We then excluded 6,427 patients, resulting in a sample size of 3,197 patients included in the analysis (mean age ± standard deviation, 46.5 ± 13.7 years; males, 38.1%; females, 61.9%). The breakdown of antidepressants prescribed for first-time treatment of MDD in Japan is shown in **Figure 2**. The most prescribed antidepressant was escitalopram (18.7%, n = 597), followed by mirtazapine (17.5%, n = 561) and sertraline (15.4%, n = 493).

### 3.2 Incidence of actionable drug-gene interactions

Phenotype frequencies for CYP2C19, CYP2B6, and CYP2D6 are summarized in **Table 1**. Additionally, the combined phenotype frequencies for CYP2C19 with CYP2B6 and CYP2C19 with CYP2D6 are detailed in **Tables 2**, **3**, respectively.

**Table 1:** Phenotype frequencies in the Japanese population.

Gene	Phenotype				References
	PM	IM	NM	UM	
CYP2C19	0.175	0.488	0.333	0.003	23
CYP2B6	0.036	0.275	0.583	0.107	24–26
CYP2D6	0.005	0.253	0.732	0.012	23

PM, Poor metabolizer; IM, Intermediate metabolizer; NM, Normal metabolizer; UM, Ultrarapid metabolizer.

**Table 2:** Phenotype frequencies for CYP2C19 combined with CYP2B6.

		CYP2B6			
		PM	IM	NM	UM
CYP2C19	PM	0.006	0.048	0.102	0.019
	IM	0.017	0.134	0.284	0.052
	NM	0.012	0.092	0.194	0.036
	UM	0.0001	0.001	0.002	0.0003

PM, Poor metabolizer; IM, Intermediate metabolizer; NM, Normal metabolizer; UM, Ultrarapid metabolizer.

**Table 3:** Phenotype frequencies for CYP2C19 combined with CYP2D6.

		CYP2D6			
		PM	IM	NM	UM
CYP2C19	PM	0.001	0.044	0.128	0.002
	IM	0.002	0.123	0.357	0.006
	NM	0.002	0.084	0.244	0.004
	UM	0.00002	0.001	0.002	0.00004

PM, Poor metabolizer; IM, Intermediate metabolizer; NM, Normal metabolizer; UM, Ultrarapid metabolizer.

**Table 4** summarizes all estimates of actionable DGIs associated with first-time antidepressant treatment for MDD in Japanese patients. Drugs with actionable PGx were prescribed as first-time treatment for MDD in 56.5% (n = 1,807) of the patients. Additionally, it was estimated that 26.4% (n = 844) of the patients required actionable therapeutic recommendations. Individual prescription rates of drugs with actionable PGx for CYP2C19, CYP2B6, and CYP2D6 were 37.0% (n = 1184), 15.4% (n = 493), and 22.4% (n = 717), respectively. The incidence rates of DGIs associated with CYP2C19, CYP2B6, and CYP2D6 were estimated to be 23.1% (n = 740), 4.8% (n = 153), and 2.8% (n = 89), respectively. The highest incidence of actionable DGIs was observed in escitalopram and CYP2C19 pairs (12.4%, n = 398). The incidence of actionable DGIs in the sertraline and CYP2C19 or CYP2B6 pairs was 11.0% (n = 352). Notably, among the antidepressants with actionable PGx, the incidence of actionable DGIs in CYP2D6 was the highest for paroxetine, but only at 1.8% (n = 56).

**Table 4:** Estimates of actionable DGIs for first-time antidepressant treatment.

Drug	Prescription rate (n = 3197), n (%)	Phenotype	Actionable DGIs incidence (n = 3197), n (%)	CPIC recommendation
CYP2C19				
Escitalopram	597 (18.7)	PM	104 (3.3)	Reduce maintenance dose by 50%
		IM	291 (9.1)	Lower maintenance dose
		UM	2 (0.1)	Alternative antidepressant
Sertraline	493 (15.4)	PM	86 (2.7)	Reduce maintenance dose by 50%
		IM	241 (7.5)	Lower maintenance dose
Amitriptyline	42 (1.3)	PM	7 (0.2)	Avoid tertiary amine
		UM	0 (0.0)	Avoid tertiary amine
Clomipramine	41 (1.3)	PM	7 (0.2)	Avoid tertiary amine
		UM	0 (0.0)	Avoid tertiary amine
Imipramine	11 (0.3)	PM	2 (0.1)	Avoid tertiary amine
		UM	0 (0.0)	Avoid tertiary amine
Trimipramine	0 (0.0)	PM	0 (0.0)	Avoid tertiary amine
		UM	0 (0.0)	Avoid tertiary amine
Subtotal	1184 (37.0)	–	740 (23.1)	–
CYP2B6				
Sertraline	493 (15.4)	PM	18 (0.6)	Reduce maintenance dose by 25%
		IM	135 (4.2)	Lower maintenance dose
Subtotal	493 (15.4)	–	153 (4.8)	–
CYP2C19/CYP2B6				
Sertraline	493 (15.4)	PM/PM	3 (0.1)	Alternative antidepressant
		PM/IM	24 (0.8)	Reduce maintenance dose by 50%
		PM/NM	50 (1.6)	Reduce maintenance dose by 50%
		PM/UM	9 (0.3)	Reduce maintenance dose by 50%
		IM/PM	9 (0.3)	Reduce maintenance dose by 50%
		IM/IM	66 (2.1)	Lower maintenance dose
		IM/NM	140 (4.4)	Lower maintenance dose
		NM/PM	6 (0.2)	Reduce maintenance dose by 25%
		NM/IM	45 (1.4)	Lower maintenance dose
		UM/UM	0 (0.0)	Higher maintenance dose
Subtotal	493 (15.4)	–	352 (11.0)	–
CYP2D6				
Paroxetine	209 (6.5)	PM	1 (0.0)	Reduce starting and maintenance doses by 50%
		IM	53 (1.7)	Lower starting dose
		UM	3 (0.1)	Alternative antidepressant
Fluvoxamine	101 (3.2)	PM	1 (0.0)	Reduce starting dose by 25–50%
Venlafaxine	71 (2.2)	PM	0 (0.0)	Alternative antidepressant
Vortioxetine	236 (7.4)	PM	1 (0.0)	50% of starting dose and the maximum dose of 10 mg
		UM	3 (0.1)	Alternative drug
Amitriptyline	42 (1.3)	PM	0 (0.0)	Avoid tricyclic use
		IM	11 (0.3)	Reduce starting dose by 25%
		UM	1 (0.0)	Avoid tricyclic use
Clomipramine	41 (1.3)	PM	0 (0.0)	Avoid tricyclic use
		IM	10 (0.3)	Reduce starting dose by 25%
		UM	0 (0.0)	Avoid tricyclic use
Imipramine	11 (0.3)	PM	0 (0.0)	Avoid tricyclic
		IM	3 (0.1)	Reduce starting dose by 25%
		UM	0 (0.0)	Avoid tricyclic
Nortriptyline	6 (0.2)	PM	0 (0.0)	Avoid tricyclic
		IM	2 (0.1)	Reduce starting dose by 25%
		UM	0 (0.0)	Avoid tricyclic
Trimipramine	0 (0.0)	PM	0 (0.0)	Avoid tricyclic use
		IM	0 (0.0)	Reduce starting dose by 25%
		UM	0 (0.0)	Avoid tricyclic
Subtotal	717 (22.4)	–	89 (2.8)	–
CYP2C19/CYP2D6				
Amitriptyline	42 (1.3)	PM/PM	0 (0.0)	Avoid amitriptyline
		PM/IM	2 (0.1)	Avoid amitriptyline
		PM/NM	5 (0.2)	Avoid amitriptyline
		PM/UM	0 (0.0)	Avoid amitriptyline
		IM/PM	0 (0.0)	Avoid amitriptyline
		IM/IM	5 (0.2)	Reduce starting dose by 25%
		IM/UM	0 (0.0)	Avoid amitriptyline
		NM/PM	0 (0.0)	Avoid amitriptyline
		NM/IM	4 (0.1)	Reduce starting dose by 25%
		NM/UM	0 (0.0)	Avoid amitriptyline
		UM/PM	0 (0.0)	Avoid amitriptyline
		UM/IM	0 (0.0)	Alternative drug
		UM/NM	0 (0.0)	Alternative drug
		UM/UM	0 (0.0)	Avoid amitriptyline
Subtotal	42 (1.3)	–	16 (0.5)	–
**Total**	1807 (56.5)	–	844 (26.4)	–

DGIs, Drug-gene interactions; CPIC, Clinical Pharmacogenetics Implementation Consortium; PM, Poor metabolizer; IM, Intermediate metabolizer; NM, Normal metabolizer; UM, Ultrarapid metabolizer.

## 4 Discussion

In this study, we estimated potential DGIs in first-time pharmacotherapy for Japanese patients with MDD. More than half of the antidepressants prescribed for the first time to patients with MDD were drugs with actionable PGx. Notably, it was estimated that one in four patients potentially met the actionable recommendations, which included dose adjustments and switching to alternative drugs. The clinical impact of DGIs in this study substantially exceeded comprehensive estimates in primary care (16, 17). Furthermore, a Danish cohort study has shown that patients with MDD have higher lifetime use of PGx drugs (somatic and psychotropic drugs) than the general population (27). These findings suggest that the benefit of PGx testing in patients with MDD is greater than that in other therapeutic areas.

The drug-gene pair with the highest incidence of actionable DGIs was escitalopram and CYP2C19 (12.4%). Asians have a higher frequency of CYP2C19 PMs than Europeans (28), particularly in the Japanese population (29). A meta-analysis by Koopmans et al. estimated that the Japanese population had the fourth highest CYP2C19 non-NM probability worldwide (66.5%) (23). In addition to this genetic characteristic, escitalopram was the most commonly prescribed antidepressant for first-time treatment of MDD in Japan, which led to the estimation that the PGx testing would be of great value. Sertraline, which has the third highest prescription rate after escitalopram and mirtazapine, may also benefit from PGx testing because it is associated with both CYP2C19 and CYP2B6 (15).

Although only few actionable DGIs related to CYP2D6 were detected in this study, CYP2D6 was the most influential pharmacological gene interacting with PGx drugs in several European population-based studies (7, 17). The main reason is that CYP2D6 PM alleles are found at a relatively high rate (5–10%) in Europeans, whereas they are rarely observed in the Japanese population (<1%) (23). In contrast, more than 40% of the East Asian population has a decreased-function CYP2D6*10 allele, which is considerably higher than that in European populations (30). Some CYP2D6*10-containing genotypes are classified as intermediate metabolizers (IMs) (31), and the probability of CYP2D6 IMs is estimated to be approximately 25% in the Japanese population (23). Although the CPIC guidelines do not provide actionable recommendations for CYP2D6 IMs with respect to fluvoxamine, mirtazapine, and venlafaxine (15), a meta-analysis has shown that there is a significant increase in exposure to these antidepressants (13), which warrants further discussion.

SLC6A4 and HTR2A genotypes have also been thought to be associated with the response to antidepressants and their side effects, although the results of relevant studies have been inconsistent (15). Therefore, no clinical recommendations have been provided in the CPIC guideline; accordingly, these genes were excluded from this analysis. If evidence is established in the future, it will be important to evaluate the impact of comprehensive pre-emptive PGx testing with inclusion of SLC6A4 and HTR2A.

The prescription patterns of first-time antidepressants in patients with MDD were considered generally consistent with the Japanese expert consensus. This recommendation states that escitalopram and sertraline are likely to be prescribed as first-line choices for patients with anxiety as the predominant symptom, and the prescription rate for these antidepressants was high in our study. Although this study did not calculate the prescription rate according to individual symptom, it is supported by the finding that more than half of patients with MDD have anxious depression (32). In contrast, trazodone, which is the third-line treatment for MDD, also showed a high prescription rate; this is likely due to its off-label use for insomnia, which may have led to overestimation.

Our study only evaluated first-time antidepressant prescriptions to focus on the promise of pre-emptive PGx testing. However, only one-third of the patients achieved remission after the initial treatment for MDD (33), and another third had treatment-resistant depression (no clinical improvement with at least two antidepressants) (34). Thus, many patients are likely to use multiple antidepressants over the course of MDD treatment by switching or combining medications. The life-time cumulative rate of PGx drugs is expected to be even higher than that of initial treatment, further increasing the potential value of PGx testing in patients with MDD.

This study had several limitations. First, the MDV is a database based on insurance claims, and records may be inaccurate (e.g., misclassification of ICD-10 coding) because of its secondary use for research purposes. In other words, some patients registered with depressive episodes (F32) in the ICD-10 may have been prescribed antidepressants for other purposes. In particular, although we restricted our analysis to prescriptions made by psychiatrists and excluded cases where these drugs were used for physical conditions such as fibromyalgia, it should be noted that not all of these patients necessarily had MDD. Second, the DPC system applies to general wards; therefore, psychiatric hospitals that do not have these were not included in the data source for this study because the DPC was not adopted. Because the data included in this study were primarily obtained from patients with MDD who were treated in hospitals with general wards, the generalizability of our findings may be limited. Third, the DPC hospitals that comprise the MDV database are responsible for acute care and include patients referred from primary care (prevalent user) in addition to those receiving first-time treatment for MDD. We adopted a new-user design to exclude prevalent users, but it was difficult to completely eliminate this factor (35). Fourth, because this study focused on patients with MDD, it was not possible to evaluate prescriptions for other indications. Some antidepressants are also recommended for the treatment of anxiety disorders (36), and pre-emptive PGx testing may be beneficial for patients who do not have MDD. However, further investigation is required to determine the clinical impact of PGx testing for patients with other conditions. Finally, potentially actionable DGIs were calculated based on prescription patterns in Japan and phenotype frequencies in the Japanese population, making it difficult to extrapolate the results to other countries. For instance, citalopram is commonly prescribed in the United States but has not been approved in Japan (37). Future studies should aim to validate these findings in larger, more diverse populations, including those from different ethnic backgrounds, and consider additional factors such as comorbidities and treatment resistance to further elucidate the clinical value of PGx testing for patients with MDD.

In conclusion, our study estimated that one in four Japanese patients with MDD who were prescribed first-time antidepressants had actionable DGIs which could have been mitigated had pre-emptive PGx testing been performed. Particularly, DGIs associated with the drug-gene pair of escitalopram, the most prescribed antidepressant in Japan, and CYP2C19, a gene with high frequency of non-NMs, may affect a large number of patients with MDD. These findings highlight the potential effectiveness of pre-emptive PGx testing in optimizing antidepressant selection and dosing.

## Data Availability

The original contributions presented in the study are included in the article/**Supplementary Material**.

## Supplementary material

Supplementary Figure 1 Study design diagram.
