# Supplementary figures and images for: Estimating the incidence of actionable drug-gene interactions in Japanese patients with major depressive disorder
Source: Front Psychiatry. 2025 Mar 27;16:1542000. doi: 10.3389/fpsyt.2025.1542000 (PMC11983551; doi:10.3389/fpsyt.2025.1542000)

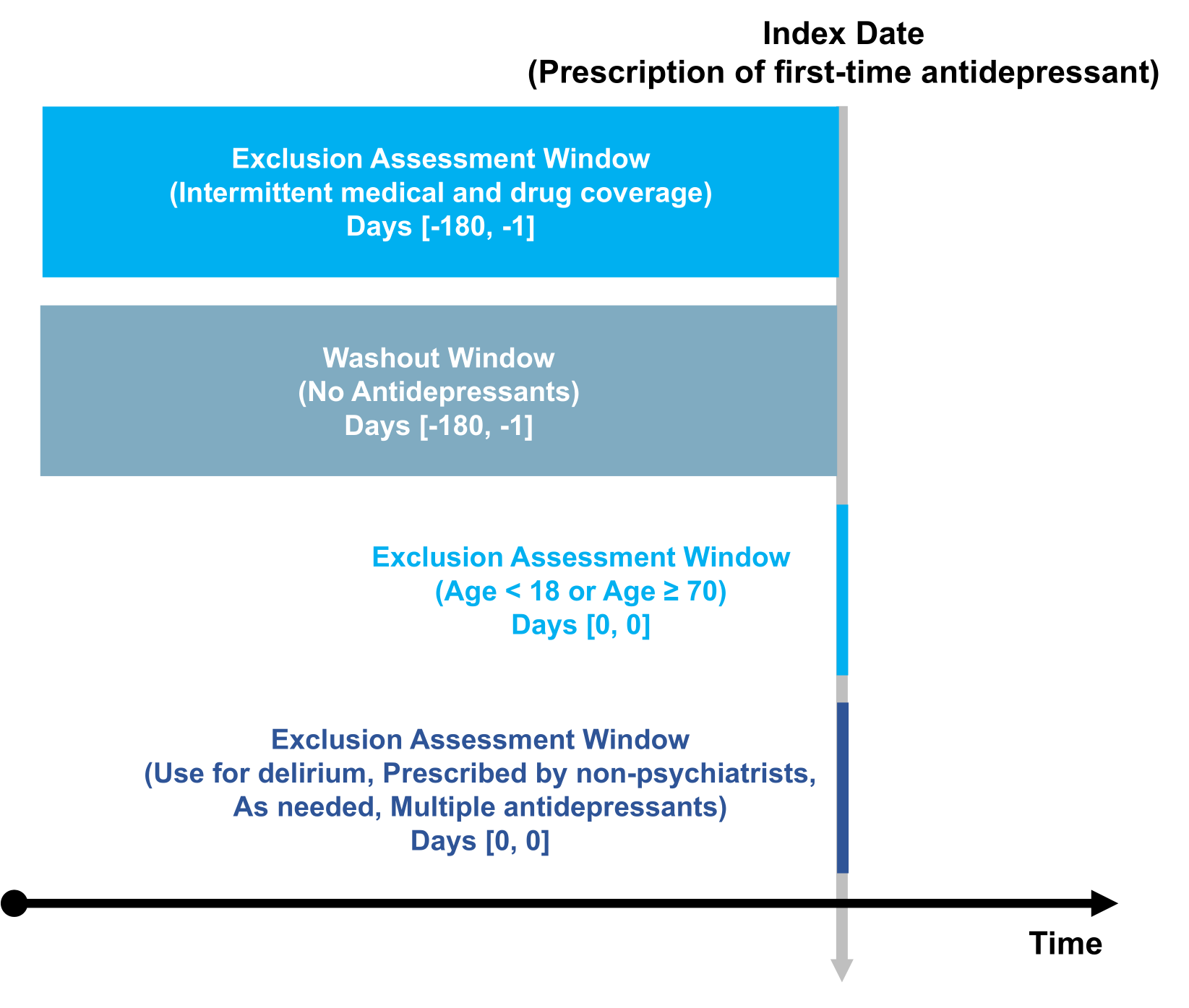

Supplement: Supplementary Figure 1 — Study design diagram. [file Image1.tiff]
